# Stellate Cell Networks in the Teleost Pituitary

**DOI:** 10.1038/srep24426

**Published:** 2016-04-18

**Authors:** Matan Golan, Lian Hollander-Cohen, Berta Levavi-Sivan

**Affiliations:** 1 Department of Animal Sciences, The Robert H. Smith Faculty of Agriculture, Food and Environment, The Hebrew University of Jerusalem, Rehovot 76100, Israel

## Abstract

The folliculostellate cells of the mammalian pituitary are non-endocrine cells that are implicated in long-distance communication and paracrine signaling, but to date, these cells have yet to be characterized in teleosts. We found that the stellate cells of the teleost pituitary share many common attributes with mammalian folliculostellate cells. By labeling of stellate cells in live preparations of tilapia pituitaries we investigated their distribution, association with other endocrine cells and their anatomical and functional coupling. In the pars intermedia, stellate cells were arranged around neuronal bundles and their processes extended into the pars distalis. Within the pars distalis, stellate cells formed close associations with FSH cells and, to a lesser degree, with GH and LH cells, suggesting differential paracrine regulation of the two gonadotrope populations. The production of follistatin by stellate cells further corroborates the notion of a paracrine role on FSH release. We also found stellate cells to form gap junctions that enabled dye transfer to neighboring stellate cells, implicating that these cells form a large-scale network that connects distant parts of the pituitary. Our findings represent the first wide-scale study of stellate cells in teleosts and provide valuable information regarding their functional roles in pituitary function.

Folliculostellate cells^1^ were identified in mammals in 1953 during the first electron microscopy (EM) investigations of the pituitary as agranular stellate cells^2^. Since then, these cells have received substantial attention in an attempt to reveal their functional importance; nevertheless, their specific role remains largely unknown^3^. A number of possible roles have been implicated for these enigmatic cells. Existing data suggest that folliculostellate cells can affect other cell types through mostly inhibitory paracrine interactions^3^,^4^,^5^, and form extensive networks in mammalian pituitaries that functionally link distant parts of the glands by gap-junction-mediated coupling^6^,^7^. Folliculostellate cells were also shown to play a role in phagocytic processes^8^.

Despite the fact that a considerable body of literature exists regarding the structure and function of fish pituitaries, folliculostellate cells were never identified in this largest group of vertebrates. While many of the classical ultrastructural studies identified non-granulated cells in various parts of the fish pituitary^9^,^10^,^11^,^12^,^13^,^14^, the failure to label them in live tissue, or even in light-microscope preparations, hindered the ability to study the general architecture and functional role of these cells in the gland. In this study we labeled stellate cells in live preparations of fish pituitaries and describe striking similarities between the teleost stellate cells and mammalian folliculostellate cells.

The hypothalamo-pituitary (HP) axis in fish conserves the major components and functions of the axis in higher vertebrates^15^, yet it exhibits several unique features: The fish pituitary is highly compartmentalized and each cell type resides in a distinct location within the gland whereas in mammals the cell types are distributed throughout the gland^11^. In teleosts prolactin, adrenocorticotropic hormone (ACTH) and thyroid-stimulating hormone (TSH) producing cells are situated in the rostral pars distalis (RPD); growth hormone (GH), luteinizing hormone (LH) and follicle-stimulating hormone (FSH) producing cells are located in the proximal pars distalis (PPD); whereas melanocyte-stimulating hormone (MSH) and somatolactin are released from cells in the pars intermedia (PI)^16^. Another unique feature of teleost pituitaries is the direct innervation of hypothalamic fibers into the endocrine tissue of the gland, releasing their content adjacent to endocrine cells or the pituitary vasculature^17^, whereas in mammals the hypothalamic axons terminate in the median eminence, from which the signals are carried through the circulation to the pituitary^11^. Last, the gonadotropes in fish produce either LH or FSH^18^ whereas mammalian gonadotropes produce both gonadotropins simultaneously^19^.

These unique features, along with the teleost basal position in the evolution of vertebrates, make fish an exceptionally valuable model to study the structure and function of the HP axis. In the current study, we exploited the ability of stellate cells to perform specific uptake of a fluorescently-labeled dipeptide in order to label them in fish pituitaries. This approach enabled us to investigate the structural and functional networks of these cells in the tilapia, an emerging model fish species and provide the first wide-scale description of these intriguing cells in fish.

## Results

**Figure 1:**
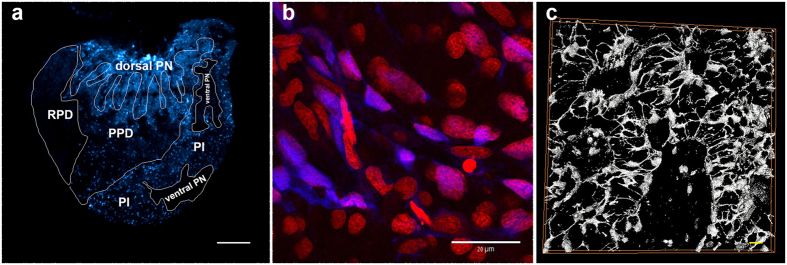
Labeling of stellate cells in the tilapia pituitary. (**a**) Sagittal section through a labeled pituitary (adult female, anterior left) shows stained cells (blue) in the dorsal PN, PI and PPD. No staining is observed in the ventral PN and in the RPD. Bar −100 μm. (**b**) Counter-staining of nuclei (red, propidium iodide) shows the angular and elongated nuclei of stellate cells (blue). (**c**) Surface rendering reveals long cytoplasmic processes that interconnect neighboring stellate cells to create a continuous structural network (width:246.0317 microns, height: 246.0317 microns, depth: 9.5673 microns. 19 slices. Bar −10 μm).

In order to label fish stellate cells we took advantage of their ability to perform specific uptake of fluorescent dipeptides^20^. In this manner, for the first time in fish, we were able to label these cells and study their unique distribution and architecture. Incubation with the dipeptide β-Ala-Lys-Nε-AMCA labeled a specific population of cells in the tilapia pituitary. Since the stained cells were not observed to form follicles at the ultrastructural level, we were not able to confidently use the term folliculostellate cells when referring to these cells. We thus chose to use the name stellate cells to indicate their stellate shape. Stellate cells were distributed within the PI as well as in the PPD but were totally absent from the RPD and ventral PN (Fig. 1a). Stained cells were relatively small, polygonal and contained an ovoid nucleus occupying most of the cell’s volume (Fig. 1b). Their long cytoplasmic processes connected them to other stellate cells, which together form a continuous structural network (Fig. 1c).

To establish whether our labeled cells express the accepted folliculostellate cells marker, we stained brains and pituitaries with antibodies raised against the folliculostellate cells marker S100B. Although specific staining was obtained in the brain (in radial glial cells) and PN, no staining was observed in the adenohypophysis (Supplementary Fig. 1). We were also unable to find specific GFAP staining in both zebrafish and tilapia adenohypophysis (data not shown).

**Figure 2:**
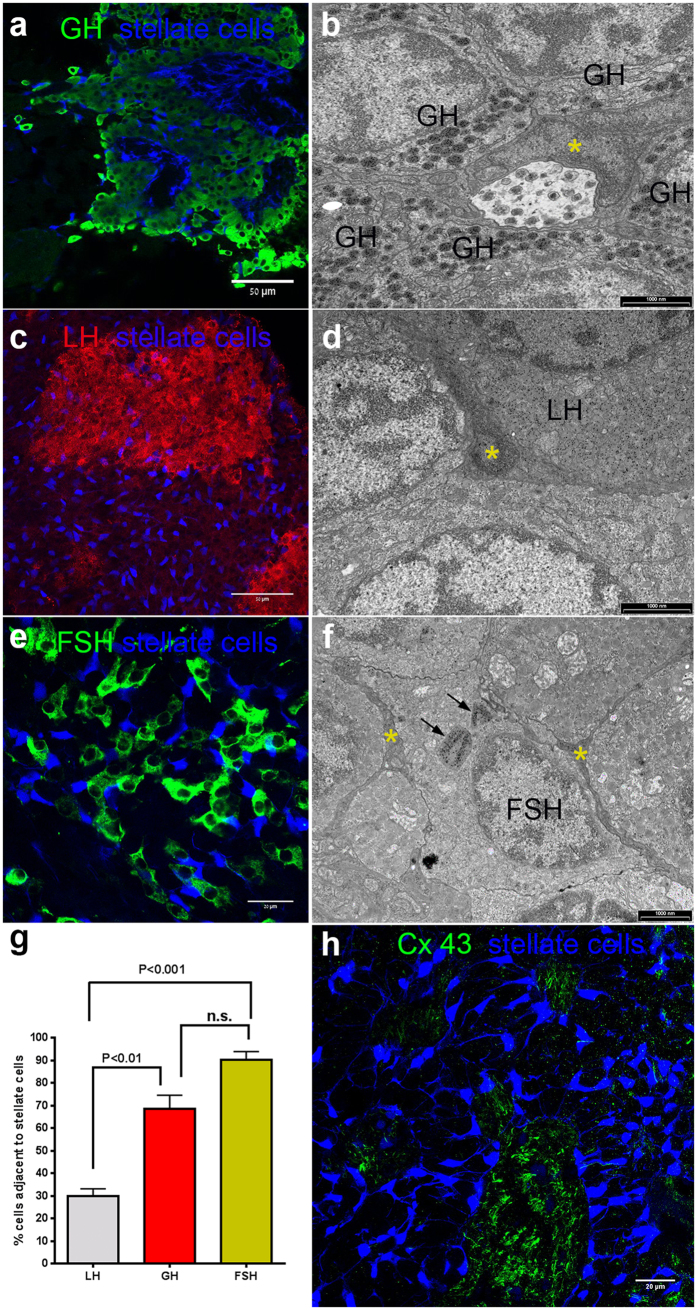
Stellate cells in the PPD and PI. (**a**) GH cell clumps (green) contain stellate cells (blue). (**b**) Stellate cells (asterisk) can be observed among immunogold-labeled somatotropes. (**c**) LH cell clumps (red) are largely devoid of stellate cells (blue). (**d**) In immunogold-labeled LH clumps, stellate cells (asterisk) can be observed mainly at the perimeter of the cell mass. (**e**) FSH cells (green) are closely associated with stellate cells (blue). (**f**) Cytoplasmic processes of stellate cells (asterisk) can be observed surrounding a FSH cell. Black arrows indicate irregular masses heavily stained with anti-tilapia FSH antibodies. (**g**) Quantification of the direct association of endocrine cells with stellate cells in the PPD. FSH and GH cells are significantly more directly contacted by stellate cells than LH cells. (**h**) A typical structure in the PI in which stellate cells (blue) surround a PN bundle in which pituicytes are immunostained for Cx 43 (green). While stellate cells do not penetrate the PN, they are situated on the perimeter of the bundle in a position that enables them to convey PN-borne messages into the pituitary parenchyma.

The distribution of stellate cells within the PPD was not uniform. GH cells in tilapia are located in the dorsal side of the pituitary and surround the PN projections. In this area many AMCA labeled cells, both from the PN and in the PPD form close contacts with the somatotropes (Fig. 2a,b). The dense LH cell clumps were rarely infiltrated by stellate cells and their projections, although in some cases close associations could be observed, especially along the perimeter of the cell aggregates (Fig. 2c,d). In contrast, FSH cells were very closely associated with stellate cells (Fig. 2e,f). Quantification of the degree of association of stellate cells with the different cell types of the PPD showed that 90% of the FSH cells form direct contacts with stellate cells (90 ± 3.6% of 138 cells counted, n = 4), whereas two thirds of the GH (68.5 ± 6.16% of 633 cells counted, n = 5) and a third of LH (30 ± 3.1% of 674 cells counted, n = 4) cells were closely associated with stellate cells (Fig. 2g). Ultrastructural investigation of stellate cells and immunogold labeled endocrine cells further corroborated these observations (Fig. 2b,d,f).

In the PI, stellate cells surrounded the neurohypophysial projections, and were connected via long processes to other stellate cells in the PI and PPD (Fig. 2h). Strong staining, probably of pituicytes, was also evident in the dorsal (anterior) PN but not within the neurohypophysial projections surrounded by the PI (posterior PN) (Figs 1a and 2h). Connexin 43 (Cx43/GJA1) staining was evident mainly on glial cell processes in the PN^21^, and no specific staining was observed on stellate cells (Fig. 2h).

**Figure 3:**
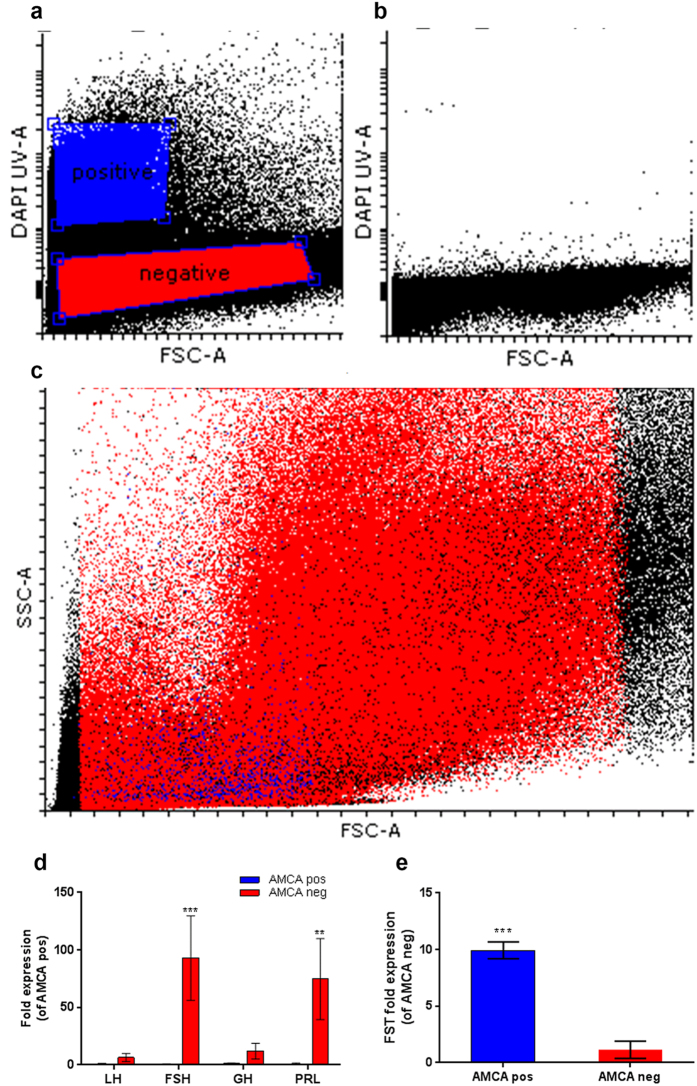
Tilapia stellate cells express follistatin. AMCA-stained (**a**,**c**) and non-stained (**b**) pituitaries were FACS-analyzed to identify and isolate stellate cells. Stellate cells (blue) are small and non-granulated (evidenced by low FSC and SSC values, (**c**). Sorted cells were analyzed by real-time PCR (**d**,**e**). While the stellate cells fraction also contained impurities from other cell types (blue bars in (**d**), follistatin is more highly expressed in stellate cells than in the general population (**e**). (***p < 0.001, **p < 0.01).

In order to further investigate the functional role of stellate cells we sorted cells from AMCA-stained pituitaries by FACS and analyzed their gene expression profiles by real-time PCR. Sorting parameters were optimized in comparison to a non-stained sample (Fig. 3a,b) and were set to exclude non-viable cells or cells with compromised membrane integrity (propidium iodide-stained cells). As expected, stellate cells comprised a relatively small (low forward-scatter (FSC) values) and non-granulated (low side-scatter (SSC) values) population when compared to the other cell types of the pituitary (Fig. 3c). Real-time PCR analysis of stained and non-stained cells revealed that the sorting procedure did not result in a completely pure stellate cells population since the stained sample also included cells that express pituitary hormones, albeit on a considerably lower level than in the general cell population (Fig. 3d). Of the genes tested, the only gene that exhibited higher expression levels in the stained population was follistatin (Fig. 3e), a known marker of folliculostellate cells^22^,^23^,^24^,^25^ and an inhibitor of FSH synthesis and release^26^,^27^,^28^,^29^,^30^,^31^.

**Figure 4:**
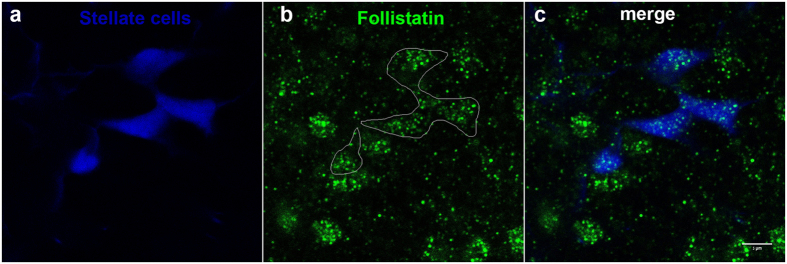
Stellate cells produce follistatin. Imunnostaining of tilapia pituitaries for follistatin reveals that stellate cells (blue) contain follistatin (green). Staining is also apparent in non-stellate cells. Bar −5 μm.

Further evidence for the expression of follistatin by stellate cells was provided by immunofluorescence. Staining tilapia pituitaries with an anti-follistatin antibody clearly labeled stellate cells (Fig. 4). Other cell types in the pituitary were also labeled, most notably the prolactin cells of the RPD (Supplementary Fig. 3). Pre-incubation of the antibody with follistatin drastically decreased staining intensity, demonstrating its specificity (Supplementary Fig. 3).

**Figure 5:**
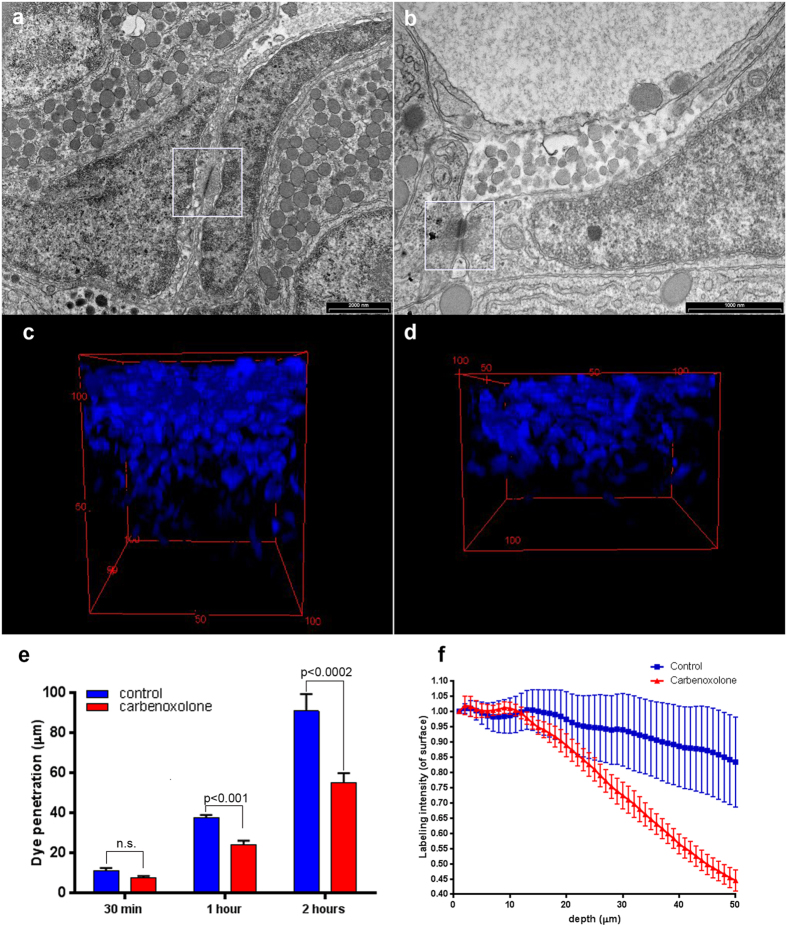
Stellate cells are connected via gap junctions to form a large scale functional network. (**a**,**b**) Two examples of TEM images displaying junctional complexes (framed) between adjacent stellate cells. (**c**,**d**) 3-D reconstruction and volume rendering of stellate cells in tilapia pituitaries incubated without (**c**) or with (**d**) the gap junction blocker carbenoxolone (100 μm). Control shows stronger staining and deeper penetration. (**e**) Application of gap-junction blockers reduces dye penetration into the tissue in a time-dependent manner (n = 8). (**f**) Staining intensity is unaffected by the treatment in the first cell layer (10 μm) but staining of deeper layers is significantly decreased by application of gap-junction blockers (n = 8).

Since folliculostellate cells in mammals are known to communicate via gap junctions, we performed ultrastructural studies of tilapia pituitaries in an attempt to identify gap junctions on the membranes of the stellate cells. At the ultrastructural level, stellate cells were seen to form junctional complexes with adjacent stellate cells (Fig. 5a,b). We were not able to observe junctional complexes on juxtaposing membranes of the stellate cells and adjacent gonadotropes or somatotropes by TEM. To further establish the functional connectivity of the stellate cells population, we utilized their ability to transfer small molecules among themselves. The fluorescent dipeptide β-Ala-Lys-Nε-AMCA used to label the stellate cells is not only taken up into the cells by specific transporters, but is also small enough to be transferred between cells through gap junctions^7^. We used this trait to determine whether the stellate cells were functionally interconnected. We incubated pituitaries with the labeled dipeptide in medium containing the gap-junction inhibitor carbenoxolone, thus exposing the superficial layer of cells to the labeled dipeptide, and examined dye penetration into the tissue using confocal microscopy. When applied at 100 μM, carbenoxolone significantly inhibited penetration of the dye into the tissue (Fig. 5c,d). Incubation time also affected penetration of the dye into the tissue, with longer incubation times resulting in deeper penetration (Fig. 5e, n = 8). Labeling intensity decreased with depth but the rate of decrease was considerably more pronounced in tissue treated with gap-junction blocker, especially within the first 50 μm. In this region, the labeling intensity remained unchanged in control pituitaries whereas in pituitaries treated with gap-junction blocker, it decreased by over 50%. No difference in dye penetration was observed within the top layer of cells (~10 μm) (Fig. 5f).

## Discussion

Folliculostellate cells have been identified in several species of mammals, based primarily on their distinct morphology in EM preparations as well as on their expression of the protein S100B. Follicular and stellate cells, probably corresponding to folliculostellate cells, have also been identified in the pituitaries of all other tetrapod classes, including avians^32^, reptiles^33^ and amphibians^34^,^35^,^36^. In teleosts, there are descriptions of the ultrastructure of non-secretory cells, especially in the RPD^9^,^12^,^13^,^14^,^37^,^38^,^39^,^40^, but also in the PPD and PI^37^,^41^. However, due to the nature of EM studies, a complete picture of the distribution of these cells is difficult to acquire, and apart from their existence and ultrastructure, virtually nothing is known about their functional importance in fish. In the absence of distinct follicular structures between adjacent stellate cells in the tilapia pituitary we were unable to define them as folliculostellate cells. Their lack of S100B expression further distinguishes them from their mammalian counterparts and we therefore chose to refer to them as stellate cells. But despite these differences, stellate cells share many attributes with mammalian folliculostellate cells, including their ultrastructure, their tendency to form a 3-dimensional network throughout the gland and their ability to uptake the labeled dipeptide.

By exploiting the specific uptake of a fluorescent dipeptide by tilapia stellate cells we could obtain a wide perspective of the distribution of these enigmatic cells in the teleost pituitary. We found clear labeling of stellate cells in the PI and PPD, but no labeling in the RPD, where prolactin and ACTH cells are located^16^. This implies that the non-secretory follicular and stellate cells that were previously described in fish, mostly in the RPD^9^,^12^,^13^,^14^,^37^,^38^,^39^,^40^, constitute a population of cells that is functionally distinct from the stellate cells identified in our work. This apparent heterogeneity in the characteristics of folliculostellate cells has been widely acknowledged in mammals, in which the folliculostellate cells population does not consist of a uniform cell type, but instead includes several subpopulations that differ in their immunophenotype and probably also in their functional role^3^. A peculiarity of fish stellate cells, which has probably hindered their identification and research, is the fact that they do not express S100B, which is the accepted marker of folliculostellate cells in mammals. Moreover, we were also unable to find glial fibrillary acidic protein (GFAP) staining in tilapia or zebrafish stellate cells, although this protein is expressed in mammalian folliculostellate cells^42^. Because the exact roles of S100B and GFAP in mammalian folliculostellate cells are unclear, it is difficult to speculate what the absence of these markers implies as to the function of fish stellate cells.

We found stellate cells to be closely associated with FSH and GH cells, but the dense LH cell aggregations contained significantly lower numbers of stellate cells. Since folliculostellate cells have been suggested to act as long-distance signal carriers to other cell types^6^,^43^, we speculated that these cells may affect the activity of FSH cells more significantly than they affect LH cells. One mode of such regulation can be paracrine, as folliculostellate cells have been shown to produce and secrete follistatin^22^,^23^,^24^,^25^. Since follistatin is a potent inhibitor of FSH expression and secretion in mammals^26^,^44^ and in fish^29^,^30^, primarily through activin binding, the close proximity of stellate cells to FSH cells places them in an appropriate position to exert this effect. To find out whether tilapia stellate cells truly express follistatin we FACS-sorted AMCA stained cells and analyzed their gene expression patterns using real-time PCR. We found the expression of the follistatin gene to be higher in the AMCA-positive cell population than in non-stained cells, implying that tilapia folliculostellate cells express this peptide. It is important to note that in our AMCA-positive FACS-sorted cell population we could still detect transcripts from other endocrine cells such as prolactin, GH and gonadotropins. This impurity probably stems from the inclusion of these cell types (attached to labeled stellate cells as “doublets”) in the stained population during the sorting procedure. We ruled out the possibility that stellate cells truly express GH, LH or FSH since immuno-staining for these hormones shows that there is no co-staining of somatotropin or gonadotropins in stellate cells. Moreover, prolactin cells are located in a region in which stained stellate cells are not observed (the RPD), meaning that the detection of prolactin in the isolated stellate cells population can only be attributed to impurity of the sorting procedure rather than to actual expression of prolactin by stellate cells. Of the genes studied, follistatin was the only transcript that was more abundant in the AMCA-positive cells than in the general cell population, implicating that this gene is in fact expressed by stellate cells. To further corroborate this finding we conducted immunofluorescence staining of tilapia pituitaries with anti-follistatin antibodies and found that stellate cells not only express the follistatin gene but also produce follistatin protein. As in mammals, the positive effects of activin, as well as the inhibitory effect of follistatin on FSH expression, are well documented in several fish species, including tilapia^28^,^29^,^30^,^31^. Therefore, the close association of stellate cells and FSH cells, coupled with the expression of follistatin by stellate cells, suggests an inhibitory role for stellate cells on FSH release. Since folliculostellate cells and follistatin also have a modulating effect on somatotropes^45^,^46^,^47^ our finding of the close association of GH and stellate cells could suggest yet another modulating role of these cells on pituitary hormone release.

At the ultrastructural level, we observed junctions between adjacent stellate cells and between stellate cells and neuronal tissue in the PI. Since mammalian folliculostellate cells have been reported to be linked by gap junctions, we expected our cells to exhibit similar connectivity. However, the junctions that we observed in the tilapia pituitary were mostly identified as tight junctions or desmosomes, by the keratin filaments seen extending toward the cytoplasm on both sides. These junctions may actually be junctional complexes made up of tight and gap junctions, since in mammalian folliculostellate cells, gap junctions are usually observed within junctional complexes between tight junctions and desmosomes^48^,^49^. It is widely accepted that in mammals, Cx 43 (gap junction A1) is expressed in folliculostellate cells^6^,^50^,^51^ but in the tilapia pituitary, Cx 43 staining was particularly evident in the pituicyte processes of the PN and not on stellate cells. This staining of the processes within the posterior PN highlights the unique organization of the stellate cells around these neuronal innervations. Stellate cells form a tight border around the perimeter of the neuronal process, and are therefore ideally located to convey signals from the neuron terminals toward the PI and further to the PPD.

We could also conclude that like mammalian folliculostellate cells, the fish stellate cells are functionally interconnected into a continuous network. Using confocal three-dimensional imaging, we found that when applied superficially, the fluorescent marker β-Ala-Lys-Nε-AMCA can be transferred between stellate cells deep into the tissue, thus proving that these cells are interconnected in a homotypic network. Gap-junction blockers significantly reduced the ability of stellate cells to transfer the dye into the tissue, resulting in inferior dye penetration and reduced staining intensity, thus implying that stellate cells coupling is gap-junction-dependent. This long-distance network is highly reminiscent of the one found in mammals^7^,^43^ and may serve to convey signals to distant pituitary regions which are not directly contacted by neuronal projections, either by direct signaling of the target cells by folliculostellate cells or by secretion of paracrine factors that specifically affect endocrine cell activity. The existence of such a network in fish pituitaries is of particular interest because the long-standing perception is that fish pituitary cells are directly innervated and regulated by hypothalamic axonal terminals^52^. Such direct regulation makes a functional stellate cells network somewhat redundant, if in fact all relevant signals can be directly delivered to their targets. However paracrine signals such as follistatin and nitric oxide that are known to be produced in folliculostellate cells^25^ are not secreted from hypothalamic axons and therefore require a different path to exert their effect. The large-scale network of stellate cells provides a suitable platform for conveying these signals throughout the teleost gland. In this respect, further research should investigate the full gene expression profile of stellate cells. The ability to label stellate cells in live tissue also presents the opportunity to perform functional studies of these cells including electrophysiological characterization, calcium imaging of their activity, interaction with other cell types in the pituitary and so forth. Such studies in fish pituitaries with all their unique features can help reveal more about the functional importance and evolutionary origins of these enigmatic cells in the vertebrate pituitary.

## Materials and Methods

### Fish husbandry

Nile tilapia (*Oreochromis niloticus*, Lake Manzala strain) were kept and bred in a recirculating water system at 26–28 °C and fed twice daily with commercial pellets (47% protein, 6% fat, Raanan Shivuk, Israel). Fish sex, weight and gonado-somatic index (GSI) are detailed for the individual experiments.

All experiments were conducted in accordance with the Animal Care and Use Guidelines of the Hebrew University and were approved by the National Research Council for Care and Use of Laboratory Animals.

### Antibodies

For immunofluorescence and immunogold labeling, we used specific antibodies raised in rabbit against tilapia growth hormone (GH)^53^, FSH^54^ and LH^55^. For immunolabeling of connexin 43 (Cx43/gap-junction A1 protein) we used a rabbit polyclonal antibody ab63851 (Abcam, Cambridge, UK). For validation of this antibody in tilapia see Supplementary Fig. 2 and Supplementary methods. For immunolabeling of follistatin we used a rabbit polyclonal antibody AAS86422C (Antibody Verify, Las Vegas, NV). Validation of this antibody in tilapia was performed by staining pituitaries with antibodies preabsorbed with recombinant human follistatin (ProSpec, Rehovot, Israel). Preabsorption results are presented in Supplementary Fig. 3. The preabsorption process was performed by incubating antibodies with (preabsorbed) or without (control) antigen (molar ratio 1:10) in antibody dilution solution (1% BSA w/v and 0.3% triton-X 100 v/v in PBS) overnight at 4 °C. These antibodies were subsequently used for immunofluorescence^56^.

### Labeling of tilapia stellate cells and gap junction blocker application

Pituitaries from adult fish (n = 8) were freshly collected into cold HEPES buffered saline (HBS, 145 mM NaCl, 5.4 mM KCl, 1.8 mM CaCl2, 1 mM MgCl2, 20 mM glucose, 20 mM Hepes, pH 7.2) and sliced in half along the longitudinal axis. Each half was then incubated in HBS containing 150 μM labeled dipeptide β-Ala-Lys-Nε-AMCA (BioTrend, Cologne, Germany)^20^, with or without 100 μM of carbenoxolone for 0.5, 1 or 2 h at 28 °C in the dark. Following incubation, pituitaries were transferred into fresh HBS (without β-Ala-Lys-Nε-AMCA) with or without carbenoxolone (according to their initial treatment) and incubated for an additional 1 h, washed 3 times in HBS, fixed in 4% paraformaldehyde for 2 h at room temperature and imaged on a confocal microscope (Ex = 405 nm, Em = 450 nm).

### Immunofluorescence

Immunofluorescence was performed on 15 μm thick cryostat sections as previously described^57^. Following dipeptide uptake pituitaries were fixed in 4% PFA overnight at 4 °C. Fixed tissue was cryoprotected in 30% sucrose, embedded in Tissue Freezing Medium (Electron Microscopy Sciences, Hatfield, PA), flash frozen in liquid N_2_ and cryosectioned to 15 μm thickness. Sections were blocked (5% normal goat serum v/v, 0.3% triton-X 100 v/v in PBS) for 1 h at room temperature. Subsequently slides were incubated with primary antibodies overnight at 4 °C. Primary antibodies against tilapia GH, FSH and LH were diluted 1:500 whereas anti Cx43 and anti follistatin antibodies were diluted 1:200 in antibody dilution solution. Secondary Alexa-conjugated antibodies were diluted 1:300 and incubated with the samples for 2 h at room temperature. All staining processes were performed in the dark.

### Image analysis

Surface rendering (for Fig. 1c) was performed by the AMIRA software (FEI, Hillsboro, OR) using the volume and isosurface rendering tools.

For determination of depth of dipeptide penetration, the thickness of the z-stack from the surface (strongest staining) to the complete disappearance of the AMCA labeling was measured. Penetration depth was measured in eight pituitary halves for each condition (with/without gap-junction blockers).

For the quantification of signal decrease with depth we used the ImageJ software^58^ to measure total AMCA fluorescence intensity on individual focal sections within the first 50 μm of the acquired z-stack in 1 μm steps.

Quantification of the proximity of gonadotropes and somatotropes to stellate cells was performed by manually counting the number of cells directly adjacent to stellate cells (mature males, GSI 0.42 ± 0.3, counts performed on 15 μm z-stacks). For the analysis 138 FSH cells (n = 4 fish, three section averaged for each fish), 633 GH cells (n = 5 fish, three section averaged for each fish) and 674 LH cells (n = 4 fish, three section averaged for each fish) were counted.

### Transmission electron microscopy (TEM)

Fresh pituitaries from adult tilapia were fixed in 4% paraformaldehyde, 0.1% glutaraldeyde in 0.1 M cacodylate buffer (pH 7.4) for 2.5 h at room temperature. The tissues were rinsed four times in cacodylate buffer and post-fixed and stained with 1% osmium tetroxide, 1.5% potassium ferricyanide in 0.1 M cacodylate buffer for 1 h. Tissues were then washed four times in cacodylate buffer, and dehydrated in increasing concentrations of ethanol, followed by 100% anhydrous ethanol and propylene oxide. Dehydrated tissues were infiltrated with increasing concentrations of agar 100 resin in propylene oxide, consisting of 25, 50, 75, and 100% resin for 16 h each step. The tissues were then embedded in fresh resin and allowed to polymerize at 60 °C for 48 h. Embedded tissues were sectioned with a diamond knife on an LKB 3 microtome and ultrathin sections (80 nm) were collected onto 300-mesh nickel grids.

The post-embedding immunocytochemical procedure was generally performed according to Castel *et al.*^59^. Grids were incubated in drops of reagent on parafilm in a wet chamber and the various steps alternated with washes in drops of Tris-buffered saline (TBS; 20 mM Tris-base, 0.9% NaCl, 0.5% BSA, 0.5% Tween-20 and 0.13% NaN3, pH 8.2). Sections were etched for 15 min in saturated aqueous sodium metaperiodate solution, followed by 60 s immersion in 1% sodium borohydride, washed five times in TBS and blocked for 1 h in 5% normal goat serum in TBS. Sections were then exposed to the primary antibodies for 1.5 h at room temperature and incubated at 4 °C for 16 h. Antibody dilutions were 1:200 for LH and GH and 1:80 for FSH. For immunogold labeling, we used a secondary 12 nm colloidal gold-conjugated goat anti-rabbit IgG (Jackson ImmunoResearch Laboratories, West Grove, PA). Incubation for 1.5 h with the secondary antibody was followed by 2 min fixation in 2% glutaraldehyde and contrasting in saturated aqueous uranyl acetate and lead citrate before air-drying. Grids were viewed with a Tecnai 12 TEM 100 kV (Philips, Eindhoven, the Netherlands) equipped with a MegaView II CCD camera and Analysis® version 3.0 software (SoftImaging System GmbH, Münstar, Germany).

### FACS sorting and real-time PCR

For the isolation of stellate cells, pituitaries from 10 adult males (body weight 73.7 ± 6.02 g; GSI 0.17 ± 0.04%) were sliced in half and incubated for 2 hours in 150 μM β-Ala-Lys-Nε-AMCA. Following uptake of the label, tissue was digested by trypsin into a single-cell suspension^60^ and filtered through a 40 μm sieve. The suspension was then sorted in a FACSAriaIII SORP sorter using a 355 laser to excite the AMCA label. Propidium iodide was added to exclude non-viable cells. A non-stained control sample was used to determine the correct parameters for gaiting the positively stained cell population (Fig. 4A,B). Three biological samples of 5,000 cells were collected for each cell population (AMCA-positive and AMCA-negative) and transcribed into cDNA using the CellsDirect cDNA Synthesis System (Life Technologies, Carlsbad, CA). Sorted cells were immediately frozen on dry ice and cDNA was prepared according to the manufacturer’s instructions. Real time PCR was performed as described previously^57^ using 1 μl of the cDNA solution as template in a 20 μl reaction volume. Primer sequences are detailed in supplementary table 1. Since a similar number of cells were analyzed in each sample, we directly compared the Ct values between the AMCA-positive and AMCA-negative populations. The data was normalized to the level of gene expression within the positive samples (in Fig. 3d) or to the level of gene expression within the negative samples (in Fig. 3e) and is presented as the fold-change between the two populations as calculated by the 2^ΔCt^ formula.

### Statistics

The results are presented as mean ± SEM. Percentage of GH, LH and FSH cells that are directly adjacent to stellate cells was compared by one-way ANOVA followed by an a-posteriori Bonferroni’s multiple comparisons test. Comparison of expression levels within the same gene was performed by student’s t-test between the positive and negative FACS-sorted samples. For comparison of dye penetration depth, a paired t-test was performed comparing the two halves of the same pituitary (effectiveness of pairing P < 0.0001). All statistical analysis was performed using the Prizm4 software (GraphPad, La Jolla, CA). Values were considered significantly different if P < 0.05.

## Additional Information

**How to cite this article**: Golan, M. *et al.* Stellate Cell Networks in the Teleost Pituitary. *Sci. Rep.* **6**, 24426; doi: 10.1038/srep24426 (2016).

## Supplementary Material

Supplementary Information
