# Supplementary material for: Stellate Cell Networks in the Teleost Pituitary
Source: Sci Rep. 2016 Apr 18;6:24426. doi: 10.1038/srep24426 (PMC4834476; doi:10.1038/srep24426)
Supplement: Supplementary Information [file srep24426-s1.pdf]

Supplementary Figures and Method

Stellate Cell Networks in the Teleost Pituitary

Matan Golan, Lian Hollander-Cohen and Berta Levavi-Sivan*

Supplementary Table 1- Primer pairs used for real-time PCR

Gene	Position	5' to 3' sequence	Accession No.
fshb	174F	CTGTCGCCCAAAGAACATCA	AF289174.1
	424R	AGGTCCCGCAGTCTGTGTTT	
lhb	44F	TGCTCCTTGCTCTGATGTTGA	AY294016.1
	226R	CCTTGGTGATGCAGTGTCCAC	
fst	499F	GTTGAACCGAAGAAGCAAGC	DQ343148.1
	909R	GACTTGGCCTTGATGCATTT	
gh	151F	TGCTCGCCCAGAGACTCTTC	M26916.1
	351R	TGGGAAACTCCCAGGACTCA	
prl	183F	CAGGAGCTGGGCTCTGAAGC	M27011.1
	481R	GCAGCTGGACCCATCTTGCT	

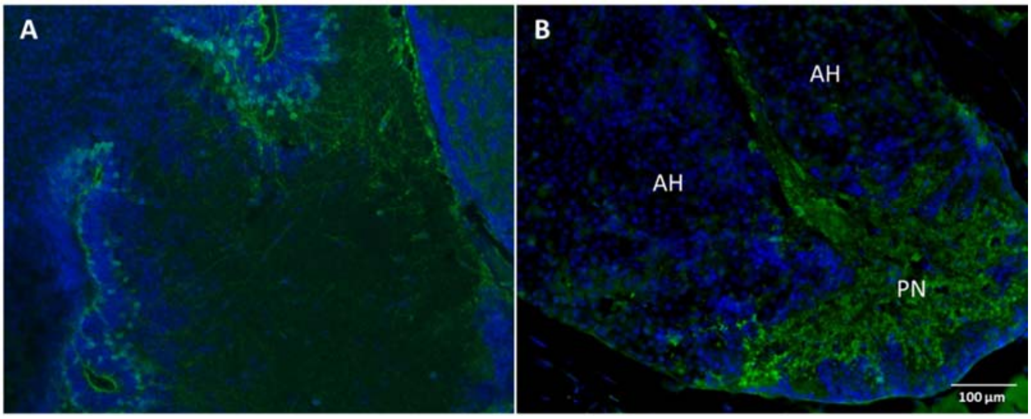

Supplementary Figure 1. Immunofluorescence staining for S100B. Brains (A) and pituitaries (B) were stained with a S100B antibody (Dako, Z0311, 1:200) that was shown to be valid in fish [1,2]. Specific staining (green) was observed in radial glial cells in the brain (**A**) and in PN projections (**B**) but not in the AH. Blue – nuclear DAPI stain

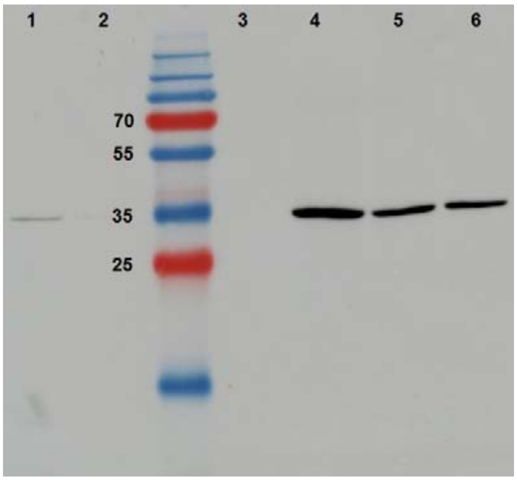

Supplementary Figure 2. Anti-Cx43 antibody validation. Western blot of tilapia pituitary (lanes 1-3) and heart (lanes 4-6, positive control) lysates. High protein levels are observed in the heart whereas in the pituitary a band is only observed in the highest concentration (lane 1). Lane 1,4 – no dilution; Lanes 2,5 – 1:10; Lanes 3,6 – 1:100.

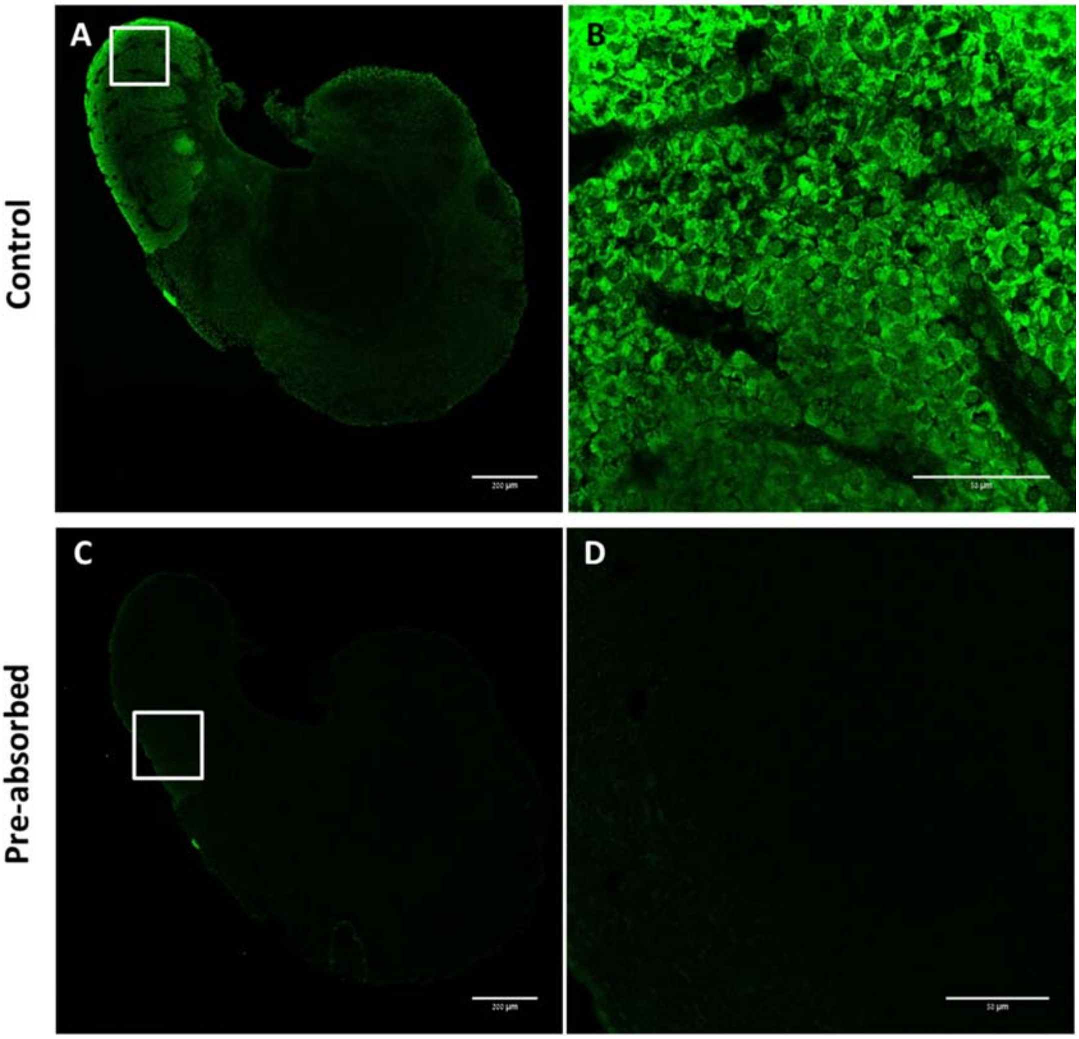

Supplementary Figure 3. Anti-follistatin antibody validation. Immunofluorescence of tilapia pituitaries was performed using anti-FST antibodies. Prior to application, antibodies were incubated without (**A** and **B**) or with (**C** and **D**) human follistatin. Strong staining is observed in the control (**A** and **B**) whereas preabsorption drastically reduced staining (**C** and **D**). **B** and **D** are magnifications of the areas framed in **A** and **C**, respectively. The sections shown are two adjacent cryosections (15 µm thick) from the same pituitary (adult male, 83g, GSI=0.27%, sagittal section, anterior-left). Bars – 200 µm in **A** and **C**, 50 µm in **B** and **D**.

Supplementary method

Western blotting was generally performed as described previously [3]. Tissues (50 mg heart or one whole pituitary) were homogenized in distilled water, frozen for 2 hours and then centrifuged for 15 minutes at 10,000xg. Supernatant was collected and directly loaded onto the gel after appropriate dilution in distilled water. Blocked membrane was probed by anti Cx43 antibody (ab63851, 1:200, Abcam, Cambridge, UK) and visualized using EZ-ECL.

References

1. Germanà, A. *et al.* Expression and distribution of S100 protein in the nervous system of the adult zebrafish (Danio rerio). *Microsc. Res. Tech.* **71,** 248–55 (2008).

2. Kroehne, V., Freudenreich, D., Hans, S., Kaslin, J. & Brand, M. Regeneration of the adult zebrafish brain from neurogenic radial glia-type progenitors. *Development* **138,** 4831–41 (2011).

3. Aizen, J., Kasuto, H., Golan, M., Zakay, H. & Levavi-Sivan, B. Tilapia follicle-stimulating hormone (FSH): immunochemistry, stimulation by gonadotropin-releasing hormone, and effect of biologically active recombinant FSH on steroid secretion. *Biol. Reprod.* **76,** 692–700 (2007).
